# Evaluation of unilateral corneal collagen cross-linking on fellow untreated eyes of patients with keratoconus

**DOI:** 10.5935/0004-2749.2022-0004

**Published:** 2022-10-19

**Authors:** Emine Akçay, Ezgi Naz Ensari Delioğlu, Meltem Ece Kars, Pervin Demir, Nurullah Çağıl

**Affiliations:** 1 Yıldırım Beyazıt University, Department of Ophthalmology, Ankara, Turkey; 2 Basaksehir Cam and Sakura City Hospital, Department of Ophthalmology, Istanbul, Turkey; 3 Bilkent University, Department of Molecular, Biology and Genetics, Ankara, Turkey; 4 Yıldırım Beyazıt University, Department of Biostatistics and Medical Informatics, Ankara, Turkey; 5 Lokman Hekim University, Department of Ophthalmology, Ankara, Turkey

**Keywords:** Corneal topography, Cross-linking reagents, Keratoconus, Photosensitizing agents, Collagen/therapeutic use, Photochemotherapy/methods, Visual acuity, Topografia da córnea, Reagentes de ligações cruzadas, Ceratocone, Fármacos fotossensibilizantes, Colágeno/uso terapêutico, Fotoquimioterapia/métodos, Acuidade visual

## Abstract

**Purpose:**

This study aimed to examine the effects of unilateral corneal collagen cross-linking treatment on visual acuity and the topographic findings of the fellow untreated eye of patients who had bilateral progressive keratoconus.

**Methods:**

Patients with progressive keratoconus who underwent cross-linking treatment were screened retrospectively. A total of 188 untreated eyes of 188 patients whose eyes were treated unilaterally with either standard or accelerated cross-linking and refused cross-linking procedure for the fellow eye were included. Visual acuity and topographic findings of the fellow untreated eyes were obtained preoperatively and postoperatively at the 1^st^, 3^rd^, 6^th^, 12^th^, 24th, 30^th^, and 36^th^ months.

**Results:**

The change over time of variables examined was similar in the untreated eyes of patients who received standard and accelerated cross-linking methods (p>0.05). At the 12^th^ month, 136 (95.8%) untreated eyes were stable according to progression criteria. Only 4 (8%) eyes were progressive at the 24th month. No progression was observed in any of the 16 patients with a 36-month follow up.

**Conclusions:**

The results showed that the fellow untreated eyes of patients with bilateral progressive keratoconus did not have significant progression rates after unilateral cross-linking treatment.

## Abstract (pt)

**Objetivo:**

Examinar os efeitos do tratamento de reticulação unilateral do colágeno corneano na acuidade visual e os achados topográficos em olhos não tratados de pacientes com ceratocone progressivo bilateral.

**Métodos:**

Foram rastreados retrospectivamente pacientes com ceratocone progressivo submetidos a tratamento de reticulação. Foram incluídos no estudo 188 olhos não tratados de 188 pacientes tratado unilateralmente com reticulação padrão ou acelerada e que recusaram o procedimento de reticulação no outro olho. A acuidade visual e os achados topográficos dos olhos não tratados foram obtidos no pré- e pós-operatório no 1º, 3º, 6º, 12º, 24º, 30º e 36º mês.

**Resultados:**

As alterações ao longo do tempo foram semelhantes para as variáveis examinadas nos olhos não tratados de pacientes tratados com métodos de reticulação padrão e acelerado (p>0,05). No 12º mês, 136 olhos não tratados (95,8%) estavam estáveis, de acordo com os critérios de progressão. Apenas quatro olhos (8%) mostraram progressão no 24º mês. Nenhuma progressão foi observada nos 16 pacientes que tiveram um acompanhamento de 36 meses.

**Conclusões:**

O estudo mostrou que os olhos não tratados de pacientes com ceratocone progressivo bilateral não apresentaram taxas de progressão significativas após o tratamento unilateral com reticulação.

## INTRODUCTION

Corneal collagen cross-linking (CXL) was first used in the 1990s for the treatment of progressive corneal ectasia. The main purpose of this treatment is to strengthen the tissue by sensitizing collagens in the connective tissue with riboflavin and forming covalent bonds with ultraviolet A (UVA) irradiation^(1)^. CXL is used mainly for the treatment of keratoconus (KC) and diseases such as pellucid marginal degeneration, pseudophakic bullous keratopathy, infectious keratitis, and post-LASIK corneal ectasia. CXL is the only treatment method that was found to stop the progression of KC^(2)^. The long-term results of many studies showed stabilization or reduction in keratometry (K) values, stabilization or increase in best-corrected visual acuity (BCVA), and reduction in topographic astigmatism and spherical equivalent (SE) after the treatment^(1,2)^. To date, many CXL methods have been introduced, including the standard method (Dresden protocol), accelerated CXL, and iontophoresis CXL. Although the results of the studies comparing these methods varied, all three methods were shown to stop KC progression^(1)^.

Some studies have observed the effects of unilateral treatment methods or drugs on fellow untreated eyes of patients with retina or glaucoma^(3,4,5,6)^. To the best of our knowledge, only Or L and Simantov I evaluated the outcomes of the fellow untreated keratoconic eye after unilateral CXL treatment, which are the continuation of each other, through a retrospective analysis^(7,8)^. In this study, we aimed to investigate the effects of unilateral CXL treatment on visual acuity and topographic findings of untreated eyes of patients who had bilateral progressive KC and refused CXL therapy for the fellow untreated eye.

## METHODS

### Subjects and inclusion

In this retrospective cohort study, more than 4000 patients with KC followed in the Keratoconus and Refractive Surgery Center at Yıldırım Beyazıt University were evaluated retrospectively. The study followed the principles of the Declaration of Helsinki and was approved by the local ethics committee.

In total, 188 untreated eyes of 188 patients who received CXL treatment for one eye and refused the treatment for the fellow eye were included in the study. The inclusion criteria for the study were as follows: (1) being diagnosed with bilateral KC, (2) having progressive KC in both eyes, and (3) being treated unilaterally with either standard or accelerated CXL. The exclusion criteria were as follows: a systemic disease, an ophthalmological disease other than KC, use of systemic or ophthalmological medications, a history of herpetic keratitis, and pregnancy.

### Treatment procedure

Of the 188 patients, 67 (35.6%) were treated with the standard technique, and 121 (64.4%) patients were treated with an accelerated method.

*Standard Technique (Dresden protocol):* After instillation of topical anesthesia with 0.05% proparacaine HCl (Alcaine 0.05%; Alcon), patients were draped. Alcohol (20%) was applied in a LASEK Funnel for 15 s, and the limbal region was protected. After 15 s, alcohol was removed using a microsurgical sponge, and the ocular surface was irrigated with a balanced salt solution. An 8-mm diameter corneal epithelium debridement was performed mechanically, and corneal thickness at the apex was measured by ultrasound pachymetry. Isotonic riboflavin (0.1% riboflavin in 20% dextran T500 solution; Meran Medicine, BNM Inc., Istanbul, Turkey) solution was dropped into the eyes with corneal thickness >400 µm, and hypotonic riboflavin (0.1% riboflavin without dextran) solution was introduced into the eyes with corneal thickness <400 µm every 2 min for 30 min. Riboflavin solution (0.1%) was applied for 30 min, followed by UVA irradiation with a wavelength of 370 nm and a power of 3 mW/cm^2^ at a 5-cm working distance for 30 min (Apollon System; Meran Medicine, Istanbul, Turkey).

*Accelerated Method:* The same steps described above were followed until epithelial debridement. After the epithelia was removed, isotonic riboflavin solution was used for the eyes with corneal thickness >400 µm, and hypotonic riboflavin solution was used for the eyes with corneal thickness <400 µm for 30 min (one drop every 2 min). UVA irradiation was performed on the central cornea for 10 min (Apollon System; Meran Medicine, Istanbul, Turkey) for an intended irradiance of 9 mW/cm^2^ at a 5-cm working distance.

After both procedures, a silicon-hydrogel bandage contact lens was applied for 5 days until complete re-epithelialization of the cornea. Topical ofloxacin drops (Exocin; Allergan Inc., Dublin, Ireland) were administered four times a day for 10 days. Tluorometholone acetate 0.1% drops (Flarex; Alcon Laboratories Inc., Mississauga, Canada) were administered topically four times a day, tapered by one drop every 2 weeks, and stopped at the end of the second month. Additionally, non-preserved artificial teardrops (Eyestil; Teka Technical Devices Industry and Trade Inc., Istanbul, Turkey) were applied six times a day for 1 month.

### Measurements

The following data were recorded preoperatively and postoperatively at the 1^st^, 3^rd^, 6^th^, 12^th^, 24^th^, 30^th^, and 36th months: uncorrected visual acuity (UCVA), BCVA, subjective cylinder refraction (Cyl), spherical equivalent (SE), slit lamp biomicroscopy findings (corneal thinning, enlarged corneal nerves, Fleisher ring, Vogt striae, and characteristic inspectional findings such as Munson and Rizutti signs), fundus examination, intraocular pressure (IOP) measurement using Goldmann applanation tonometry, and corneal topography outcomes including minimum keratometry (Kmin), maximum keratometry (Kmax), mean keratometry (Kmean), keratometry at the apex (Kapex), central corneal thickness (CCT), corneal thickness at the apex (CTA), and thinnest corneal thickness (TCT) using Sirius topographer (Costruzione Strumenti Oftalmici; Florence, Italy). The untreated eyes in stages 1 (early) and 2 (moderate) according to the Amsler Krumich classification were defined as group 1, and the untreated eyes in stage 3 (severe) were categorized as group 2^(9)^. Progression was defined as meeting one or more of the following criteria: an increase in the Kmax of at least 1.0 diopter (D), corneal thinning of at least 30 µm, or an increase in topographic astigmatism of at least 1.0 D in 6 months^(10)^.

### Statistical analysis

The Shapiro- Wilks test was used to evaluate the suitability of the variables examined in the study to the normal distribution. The mean ± standard deviation and median (minimum and maximum) values were used for all numerical variables. The categorical variables were presented as number (n) and percentages (%).

The nonparametric analysis of longitudinal data in factorial experiments - nonparametric tests for the F1-LD-F1 design was performed to test the group (standard, accelerated), time effects, and interaction. The results obtained from the F1-LD-F1 design were given with analysis of variance-type statistics (n<200). Pairwise comparisons were interpreted with the values of the relative treatment effect. The one-sample proportion test was used to assess whether the deterioration rates obtained from the study dataset is different from the known proportion (25%). Results of appropriate statistical test (Pearson Chi square test, Fisher’s exact test, and continuity correction test) were evaluated to examine whether a difference exists between categorical variables.

For statistical analysis and calculations, IBM SPSS Statistics for Windows, version 21.0 (IBM Corp., Armonk, NY, USA) and MS-Excel 2007 programs were used. The nparLD package in the R program was used for the analysis of the F1-LD-F1 design. Significance was accepted as p<0.05.

## RESULTS

In total, 188 fellow untreated eyes of 188 patients diagnosed with progressive bilateral KC and treated with CXL unilaterally were examined retrospectively. The mean age of the patients was 24.68 ± 7.10 years, and 81 (43.1%) patients were female. The average follow-up period was 17.8 ± 10.5 months.

Specifically, 20 (33.0%) patients were under 20 years old, 84 (44.7%) were between 21- 29 years old and 42 (22.3%) were >30 years old. The distribution of patients in terms of age, sex, treatment modality, laterality, and Amsler classification is shown in table 1.

**Table 1:** Distribution of individuals in specifed variables

	n (%)		n (%)
Age		Laterality	
<20	62 (33.0)	Right	102 (54.3)
21- 29	84 (44.7)	Left	86 (45.7)
≥30	42 (22.3)	Amsler-Krumeich classification of untreated eyes	
Sex		1	162 (86.2)
Female	81 (43.1)	2	22 (11.7)
Male	107 (56.9)	3	4 (2.1)
Method			
Standard technique	67 (35.6)		
Accelerated technique	121 (64.4)		

**Figure 1:**
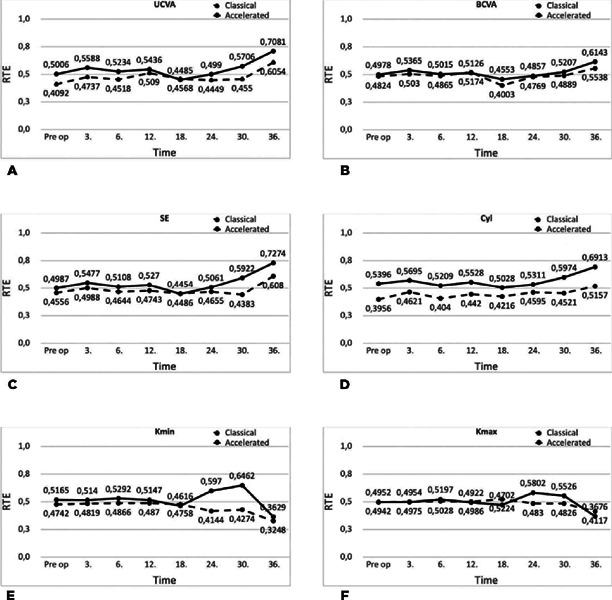
Variation of the relative effects of UCVA, BCVA, SE, Cyl, Kmin, and Kmax values of the untreated fellow eyes in classical and accelerated treatment groups according to the evaluation time.

BCVA, best-corrected visual acuity; CCT, central corneal thickness; Cyl, cylinder refraction; Kapex, keratometry at the apex; Kmax, maximum keratometry; Kmean, mean keratometry; Kmin, minimum keratometry; SE, spherical equivalent.

**Figure 2:**
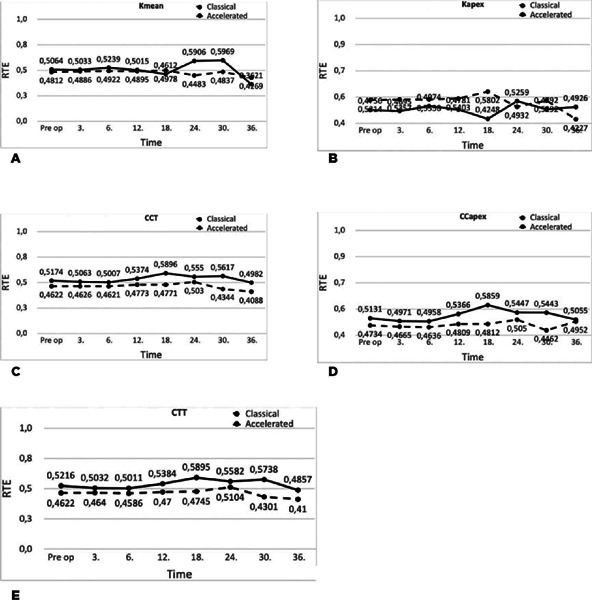
Variation of the relative effects of Kmean, Kapex, CCT, CCapex, and CTT values of the untreated fellow eyes in classical and accelerated treatment groups according to the evaluation time.

The change over time for the variables examined was similar in the untreated eyes of the patients who received standard and accelerated CXL treatment (Table 2, Figures 1 and 2). The interaction effect was not significant in all variables (p>0.05). When the difference was examined for the related variables among CXL methods independently of the evaluation times, Cyl values were the only significantly different variable in the untreated eyes (F=5.614, p=0.018). The Cyl values of the untreated eyes were higher in the accelerated group than in the standard group, with a relative impact of 0.563 and 0.444, respectively (Table 3). The values of other variables of the untreated eyes were not significantly different in either method (p>0.05).

**Table 2:** Comparison of the indicated variable values by group and evaluation times

Variables	Method		Time		Method *Time	
	F	p	F	p	F	p
UCVA	1.418	0.234	2.514	0.064	0.239	0.847
BCVA	0.422	0.516	2.054	0.090	0.138	0.961
SE	1.669	0.196	3.101	0.024*	0.430	0.740
Cyl	5.614	0.018*	1.452	0.228	0.198	0.887
Kmin	1.701	0.192	2.106	0.116	1.033	0.362
Kmax	0.027	0.869	0.952	0.395	0.336	0.742
Kmean	0.260	0.611	0.939	0.399	0.605	0.563
Kapex	0.552	0.458	0.200	0.865	0.705	0.525
CCT	1.595	0.207	0.406	0.718	0.164	0.897
CTA	0.925	0.336	0.224	0.849	0.169	0.890
CTT	1.694	0.193	0.472	0.668	0.203	0.862

BCVA= best-corrected visual acuity; CCT= central corneal thickness; CTA= corneal thickness at the apex; Cyl= cylinder refraction; Kapex= keratometry at the apex; Kmax= maximum keratometry; Kmean= mean keratometry; Kmin= minimum keratometry; SE= spherical equivalent; TCT= thinnest corneal thickness; UCVA= uncorrected visual acuity.

Kapex, keratometry at the apex; Kmax, maximum keratometry; Kmean, mean keratometry; Kmin, minimum keratometry; SE, spherical equivalent; TCT, thinnest corneal thickness; UCVA, uncorrected visual acuity.

**Table 3:** Relative effects of the method and time (RTE)

	Variables										
	UCVA	BCVA	SE	Cyl	Kmin	Kmax	Kmean	Kapex	CCT	CTA	CTT
Method											
Classical protocol	0.476	0.489	0.482	0.444	0.447	0.487	0.476	0.521	0.461	0.477	0.460
Accelerated protocol	0.544	0.516	0.544	0.563	0.518	0.497	0.506	0.480	0.533	0.528	0.534
Time											
Preop	0.455	0.490	0.477	0.468	0.495	0.495	0.494	0.503	0.490	0.493	0.492
Postop 3 month	0.516	0.520	0.523	0.516	0.498	0.496	0.496	0.503	0.484	0.482	0.484
Postop 6 month	0.488	0.494	0.488	0.462	0.508	0.511	0.508	0.517	0.481	0.480	0.480
Postop 12 month	0.526	0.515	0.501	0.497	0.501	0.495	0.495	0.509	0.507	0.509	0.504
Postop 18 month	0.453	0.428	0.447	0.462	0.469	0.496	0.480	0.502	0.533	0.534	0.532
Postop 24 month	0.472	0.481	0.486	0.495	0.506	0.532	0.519	0.510	0.529	0.525	0.534
Postop 30 month	0.513	0.505	0.515	0.525	0.537	0.518	0.540	0.504	0.498	0.495	0.502
Postop 36 month	0.657	0.584	0.668	0.603	0.344	0.390	0.395	0.458	0.453	0.500	0.448

Preop= preoperative; Postop= postoperative; BCVA= best-corrected visual acuity; CCT= central corneal thickness; CTA= corneal thickness at the apex; Cyl= cylinder refraction; Kapex= keratometry at the apex; Kmax= maximum keratometry; Kmean= mean keratometry; Kmin= minimum keratometry; SE= spherical equivalent; TCT= thinnest corneal thickness; UCVA= uncorrected visual acuity.

According to the evaluation times, independently of the CXL methods, only SE values were found significant (F=3.101; p=0.024). At the 3^rd^ and 36^th^ months, the SE values of the untreated eyes were higher than the values obtained from other evaluation times. Other variable were not significantly different (p>0.05) (Figure 1, Table 2).

The time-dependent worsening rates of each of the determined progression criteria and BCVA of the untreated eyes were examined to determine whether they were lower or higher than the expected progression rates (i.e., 25%) reported in the literature (Table 4). At the 30th month, the worsening rate of the untreated eyes in group 1 under Kmax criteria was 13.0%, which was similar to the 25% reported in the literature (z=1.324; p=0.093). At the 36th month, the progression of all 16 untreated eyes was halted. The worsening rates at the 3^rd^, 6^th^, 12^th^, 18^th^, and 24^th^ months were significantly lower than 25% (p<0.001).

**Table 4:** Worsening rates of the untreated eyes in Amsler group 1 according to the determined criteria.

	Postop 3 month	Postop 6 month	Postop 12 month	Postop 18 month	Postop 24 month	Postop 30 month	Postop 36 month
Kmax%	3.7	1.2	3.5	3.7	7.7	13.0	0.0
Z; p	-6.260;	-7.220;	-5.883;	-3.614;	-2.882;	-1.324;	-
	<0.001*	<0.001*	<0.001*	<0.001*	<0.001*	0.093	
Kmax kmin%	0.0	0.0	0.7	0.0	0.0	0.0	0.0
Z; p	-	-	-6.661;	-	-	-	-
			<0.001*				
Ccapex%	0.6	2.3	2.8	7.4	3.8	4.3	0.0
Z; p	-7.167;	-6.868;	-6.078;	-2.986;	-3.523;	-2.287;	-
	<0.001*	<0.001*	<0.001*	0.001*	<0.001*	0.011*	

Postop= postoperative

When patients who reached the 12th month control were analyzed, the untreated eyes of 6 (4.2%) patients were worsening, but 136 (95.8%) were not. At the 24th month, four untreated eyes (8%) were worsening, and the rest (n=46, 92%) were stable. No progression was observed in any of the untreated eyes (0%) of 16 patients who underwent 36^th^ month control.

The difference in sex and age group distribution was also examined. No significant difference was found in the variables of the untreated eyes, except for BCVA, between preoperative and postoperative 12th month control in women (Table 5). For the untreated eyes of patients aged <20 years, progression stopped at 93%-97.9%, and the improvement rate in BCVA was 84.8% at the 12th month. Again, the rates of halting progression ranged from 83.3% to 100% at the 24th month and 100% at the 36th month in the untreated eyes.

**Table 5:** Distribution of worsening or improving rates in BCVA of the untreated fellow eyes at 12th and 30th months according to sex

	BCVA			
	Preop-postop 12 month		Preop-postop 30 month	
	Worsening n (%)	Halting n (%)	Worsening n (%)	Halting n (%)
Sex				
Male	9 (10.8)	74 (89.2)	2 (12.5)	14 (87.5)
Female	17 (28.8)	42 (71.2)	4 (57.1)	3 (42.9)
	c^2^=6.293; p=0.012*^(a)^		p=0.045*^(b)^	

Preop, preoperative; Postop, postoperative

^(a)^ It is the result of the continuity correction test.

^(b)^ It is the result of the Fisher exact test.

## DISCUSSION

In this study, there was not significant progression on the fellow untreated eyes of patients with bilateral progressive KC after unilateral CXL treatment. Most studies have reported that CXL-treated eyes had better improvement than untreated eyes and they focused on the treated eyes. However, these studies did not discuss the progression of untreated eyes. O’Brart et al. evaluated 22 of 24 patients with early/moderate KC who had completed 18-months of follow-up. One eye of each of the 22 patients was randomly selected, treated with CXL, and compared with the untreated contralateral eye of each of the 22 patients as a control group. No significant difference was found in the UCVA, BCVA, Cyl, corneal pachymetry, Kmean, 3-mm and 5-mm K, Kapex, or mean simulated astigmatism between the two groups. The worsening rate of all parameters and evidence of progression was 14% in three untreated eyes^(11)^.

Kim et al. found insignificant worsening in BCVA and Kmax in the untreated eyes of their patients. The improvement of visual acuity, Kmax, Kmean, and corneal astigmatism in the 12th month was better in the treated eyes than in the fellow untreated eyes^(12)^. Thus, improvement was noted in the untreated eyes. Even though the follow-up period was 5 years, it is unlikely that a precedent can be set for a large group because there were nine samples. Coskunseven et al. also compared the treated eyes and their fellow untreated eyes of 19 patients. After 9 months follow-up, no significant changes were found in clinical parameters such as SE, Cyl, Kmax, and CCT, except for UCVA and BCVA^(13)^. All these studies concluded that CXL treatment was effective on the treated eyes, but they did not comment on the progression in the untreated eyes.

According to the criteria of progression accepted as a reference, the untreated eyes of 95.8% of the patients who followed up for 12 months, 92% of those followed up for 24 months, and 100% of patients who completed 36 months of follow-up did not show progression.

In the course of time, the variables in the untreated eyes were not affected by the procedure/operation performed, whether it was accelerated or standard. O’Brart et al., Kim et al., and Coskunseven et al. used the Dresden protocol, and the untreated eyes comprised the control group, as in our study^(11,12,13)^. In studies in which CXL treatment was performed by the accelerated method, the control groups did not consist of contra-lateral eyes, so we did not find a study similar to ours comparing the fellow untreated and treated eyes using the accelerated method. The comparison of the CXL methods did not reveal any significant difference in any other variables except for the Cyl value in the untreated eyes. This finding suggests that this single parametric change may be caused by the measurement differences in the fixation problems and irregular astigmatism of patients with KC due to the placement of the cone.

In addition, none of the variables showed any significant change over time, except for SE in the untreated eyes. The change in SE was determined at the 3^rd^ and 36^th^ months. The possible reason was that the effect of treatment just began in the 3^rd^ month, and in the 36^th^ month, it was due to the high number of cases that did not complete the follow-up.

The Amsler classification provides information about the condition of the untreated eyes at the time of KC diagnosis. The progression expectation of the untreated eye-which was reported as 25% after unilateral treatment of the fellow eye-was significantly similar with only Kmax at the 30th month^(14,15)^. In patients with 36-month follow-up, the progression of all untreated eyes halted, and the progression rates did not reach 25% in any of the 3^rd^, 6^th^, 12^th^, 18^th^, 24^th^, or 36^th^ month controls. The follow-up results of the untreated eyes of patients with bilateral KC treated unilaterally showed that there was not always a need to perform CXL on the untreated eyes before concrete evidence of progression.

The probability of progression in young patients with KC was reported to be high^(15)^. Chatzis found that 52 (88%) of 59 kerotoconic eyes were progressive in children and adolescent within 12 months^(16)^. Or L showed a slight decrease in UCVA, which is not significant in fellow untreated eye during 5 years of follow-up. However, the BCVA, average keratometry, and maximum keratometry remained stable^(7)^. Simantov et al. reported that 8 of the 30 untreated eyes deteriorated and underwent CXL treatment, whereas only one treated eye (3.33%) required an additional CXL^(8)^.

Although a very high proportion of young people were included in the age distribution for the variables examined, no significant difference was found for worsening or halting. In the analysis of the parameters accor ding to sex, BCVA significantly worsened in women, but there was no worsening of Kmax, Kmax-Kmin, CTA, or CTT in the untreated eyes at the 12th month control. This occurred because BCVA is not an objective and reliable evaluation criteria, and it can be different in every measurement, even after several minutes during the same examination in patients with KC.

Similar articles reported the healing effects of unilateral medical or surgical treatments on the untreated eyes detected incidentally in retina and glaucoma patients, but some studies revealed that the untreated eyes were not affected. Some hypotheses such as the ophthalmotonic consensual reaction and systemic absorption-dependent effect were suggested to explain the mechanism of glaucoma and anti-VEGF, respectively^(6,17)^. Bakbak et al. reported that bevacizumab affected the untreated eyes with diabetic macular edema by systemic absorption^(4)^. A few studies have reported similar effect for ranibizumab^(18,19)^. However, Bakri et al. reported that ranibizumab could neither be detected in the systemic circulation nor in the untreated eye after 0.5 mg intravitreal injection^(20)^.

Although KC was previously defined as a noninflammatory disease, recent studies have found inflammatory cytokines in the tear fluid of patients with KC^(21,22)^. Kolozsvari et al. evaluated 26 eyes of 23 patients in a 12-month follow-up study and examined the changes in inflammatory mediators in the tears before and after CXL. They found alterations of the mediators in the tears of patients with KC that can explain the effect of CXL^(23)^. We think that all organs and tissues of the human body are in communication with each other; thus, biochemical inflammatory markers may inform the fellow untreated eye about the unilateral CXL treatment.

The study is limited by its retrospective design, absence of patients with non-progressive KC, shorter follow-up period, and lack of inflammatory biomarker analysis to prove our hypothesis. However, no progression was observed in the untreated eyes of patients with bilateral KC who refused CXL treatment for the fellow untreated eye inspiringly.

In conclusion, the fellow untreated eyes of patients with bilateral progressive KC did not have significant progression rates after unilateral CXL treatment, and there was not always a need to perform the procedure on the second eye. Therefore, prospective randomized studies including inflammatory marker levels are warranted to explain underlying mechanisms and identify biochemical events.
